# α-Mangostin remodels visceral adipose tissue inflammation to ameliorate age-related metabolic disorders in mice

**DOI:** 10.18632/aging.102512

**Published:** 2019-12-06

**Authors:** Dan Li, Qianyu Liu, Xiuqiang Lu, Zhengqiu Li, Chunming Wang, Chung-Hang Leung, Yitao Wang, Cheng Peng, Ligen Lin

**Affiliations:** 1 State Key Laboratory of Southwestern Characteristic Chinese Medicine Resources, School of Pharmacy, Chengdu University of Traditional Chinese Medicine, Chengdu, China; 2 State Key Laboratory of Quality Research in Chinese Medicine, Institute of Chinese Medical Sciences, University of Macau, Taipa, Macau, China; 3 Fuqing Branch of Fujian Normal University, Fuzhou, China; 4 School of Pharmacy, Jinan University, Guangzhou, China; 5 State Key Laboratory of Drug Research, Shanghai Institute of Materia Medica, Chinese Academy of Sciences, Shanghai, China

**Keywords:** α-mangostin, aging, adiposity, adipose tissue inflammation, macrophage

## Abstract

Low-grade chronic adipose tissue inflammation contributes to the onset and development of aging-related insulin resistance and type 2 diabetes. In the current study, α-mangostin, a xanthone isolated from mangosteen (*Garcinia mangostana*), was identified to ameliorate lipopolysaccharides-induced acute adipose tissue inflammation in mice, by reducing the expression of pro-inflammatory cytokines and chemokines. In a cohort of young (3 months) and old (18–20 months) mice, α-mangostin mitigated aging-associated adiposity, hyperlipidemia, and insulin resistance. Further study showed that α-mangostin alleviated aging-related adipose tissue inflammation by reducing macrophage content and shifting pro-inflammatory macrophage polarization. Moreover, α-mangostin protected the old mice against liver injury through suppressing the secretion of microRNA-155-5p from macrophages. The above results demonstrated that α-mangostin represents a new scaffold to alleviate adipose tissue inflammation, which might be a novel candidate to treat aging-related metabolic disorders.

## INTRODUCTION

According to the World Health Organization data, the proportion of people aged 60 years old or over will double from about 12% to 22%, and the absolute number is expected to increase from 900 million to 2 billion by 2050. Meanwhile, aging is a common risk factor of many diseases, such as cardiovascular diseases, type 2 diabetes, neurodegenerative diseases and cancer [1]. Global aging has become one of the biggest social and economic challenges. Instead of simply extention of lifespan, more and more attention is turning to prolong ‘health span’. Thus, there is a desperate demand for effective and safe candidates to prevent and treat aging-related complications.

Aging is associated with adiposity, ectopic fat distribution, and chronic low-grade adipose tissue inflammation, which result in the onset and development of insulin resistance and related metabolic disorders [2]. Adipose tissue dysfunction results in dysregulated release of fatty acids and secretion of adipose tissue-derived hormones and microRNAs (miRNAs), which in turn influence other organs, such as liver and skeletal muscle [3]. Based on several large epidemiologic studies of older adults, the ‘inflamm-aging’ theory considers chronic inflammation as the common pathological basis for aging and aging-related metabolic diseases [4, 5]. Adipose tissue has been considered as the major contributor to the chronic, low-grade inflammation during aging [3]. Adipose tissue comprises of mature adipocytes and stromal vascular fraction (SVF), the latter mainly comprises of macrophages [6]. Accumulating evidence suggests that adipose tissue macrophages (ATMs) are a major pathogenic factor of aging and aging-related metabolic diseases [7, 8]. Classically activated macrophages (M1) contribute as the main source of pro-inflammatory cytokines, and are positively associated with obesity and insulin resistance; while alternatively activated macrophages (M2) secrete anti-inflammatory cytokines, and are associated with insulin sensitivity and lean phenotype [7, 9]. The profile of ATMs shifts towards a pro-inflammatory state in aged adipose tissue, as reflected by an increase of the ratio of M1/M2 macrophages [8]. Therefore, interventions preventing the infiltration and pro-inflammatory polarization of ATMs might be promising ways to treat aging-related metabolic disorders.

α-Mangostin (α-Man, Supplementary Figure 1A) is the main xanthone from the fruit hull of mangosteen (*Garcinia mangostana*). The anti-inflammatory property of α-Man has been widely reported, in both *in vitro* cell models, such as murine or human macrophages, adipocytes, and human adipocytes exposed to macrophage-conditioned media (CM) [10, 11], and *in vivo* animal models, such as carrageenan-induced paw edema in ICR mice and collagen-induced arthritis in DBA/1J mice [12, 13]. α-Man was reported to improve glucose uptake and inhibit differentiation in adipocytes [14]. In high-fat diet (HFD)-treated mice and rats, α-Man attenuates hepatic steatosis, insulin resistance and obesity [15, 16]. However, little is known about the role of α-Man in aging-related metabolic disorders. The current study was designed to evaluate the role of α-Man in remodeling visceral adipose tissue inflammation in LPS-induced or aging mice. This study supplies α-Man as a new candidate for alleviating adipose tissue inflammation, which might benefit the treatment of obesity, hyperlipidemia, and liver injury for the elder.

## RESULTS

### α-Man treatment reduces cytokines and chemokines levels in epididymal white adipose tissues (eWAT) from LPS-treated mice

**Figure 1:**
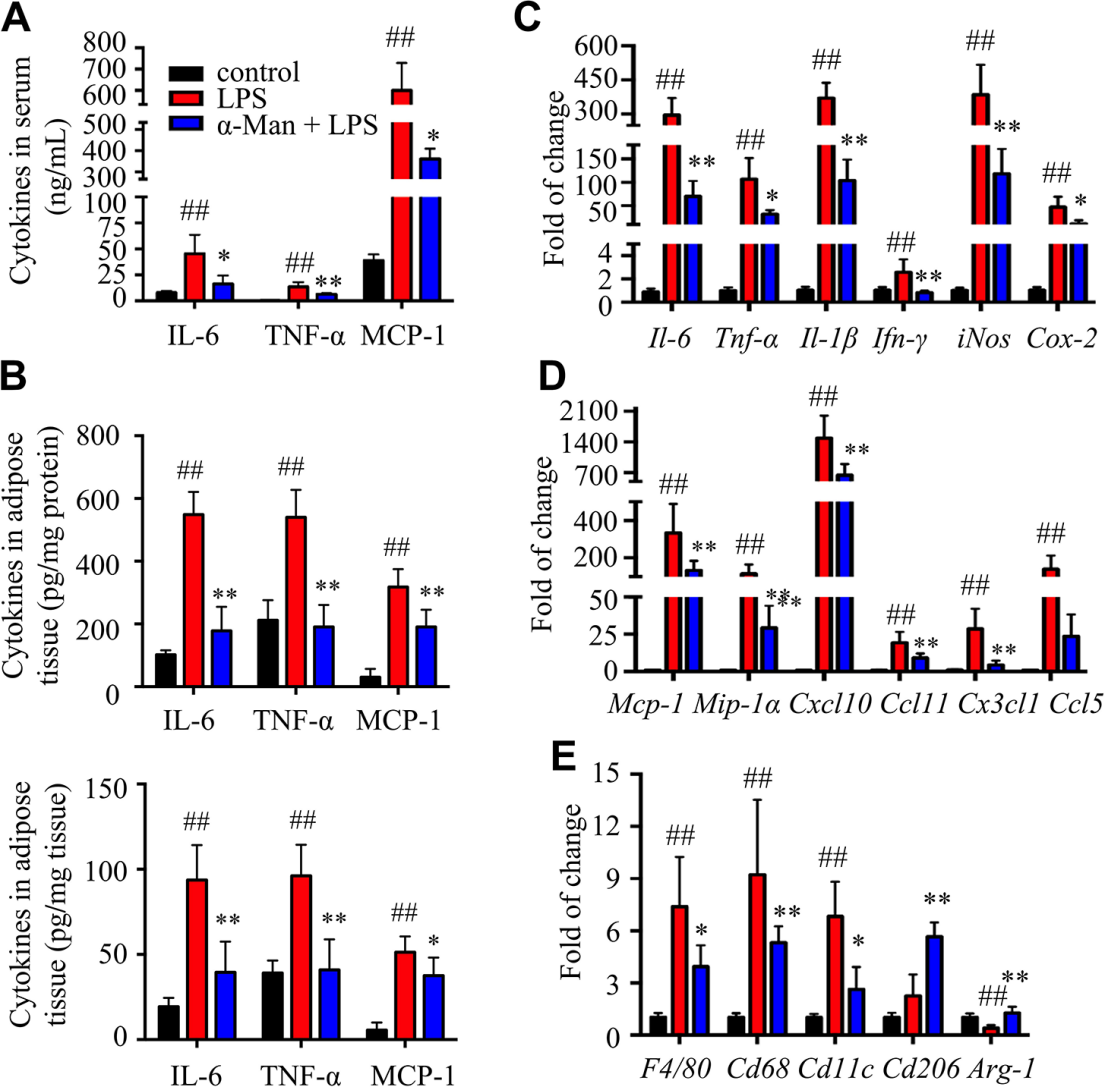
**α-Man ameliorates inflammatory responses in eWAT from LPS-treated mice.** (**A**) The serum levels of IL-6, TNF-α and MCP-1 were determined by ELISA kits. (**B**) The levels of IL-6, TNF-α and MCP-1 in eWAT were determined by ELISA kits. (**C**) qRT-PCR analyses for pro-inflammatory cytokines expression in eWAT. (**D**) qRT-PCR analyses for chemokines in eWAT, including *Mcp-1*, *Mip-1α*, *Cxcl10*, *Ccl11*, *Cx3cl1* and *Ccl5*. (**E**) qRT-PCR analyses for macrophage markers in eWAT, including *F4/80*, *Cd68*, *Cd11c*, *Cd206* and *Arg-1*. Data are expressed as means ± SD (*n* = 5). ^##^*P* < 0.01, LPS vs. control, ^*^P < 0.05, ^**^P < 0.01, α-Man + LPS vs. LPS.

Lipopolysaccharide (LPS) is a component of the outer envelope of all gram-negative bacteria, which is widely used to induce pro-inflammatory responses in adipose tissue [17]. To determine the effect of α-Man in improving adipose tissue inflammation, LPS induced acute inflammation mice model was implemented (Supplementary Figure 1B). Pre-treatment of α-Man (10 mg**/**kg/d) for 5 days didn’t affect the body weight of mice (Supplementary Figure 1C). Four hours after LPS injection, the pro-inflammatory cytokine levels, including interleukin-6 (IL-6), tumor necrosis factor-α (TNF-α) and monocyte chemoattractant protein-1 (MCP-1), were increased in serum from LPS-treated mice, compared with those from the vehicle control mice; whereas these inflammatory cytokine levels were significantly reduced in serum from α-Man pre-treated mice (Figure 1A). White adipose tissue (WAT) is distributed throughout the body; each depot has specific characteristics. The inflammation in epididymal WAT (eWAT) promotes insulin resistance and metabolic disorders [18]. Thus, we further evaluated the inflammatory responses in eWAT. Consistently, α-Man reversed LPS-induced increases of cytokines in eWAT, assessed by ELISA kits (Figure 1B) and qRT-PCR (Figure 1C). These results were in agreement with the effects of α-Man on LPS-induced RAW264.7 macrophages *in vitro* (Supplementary Figure 2A–2D).

Chemokines are a superfamily of small proteins to induce macrophage migration, which play a crucial role in immune and inflammatory reactions [19]. Next, qRT-PCR was performed to assess the expressions of chemokines in eWAT. The mRNA levels of chemokines, including *Mcp-1*, macrophage inflammatory protein-1α (*Mip-1α*), C-X-C motif chemokine ligand 10 (*Cxcl10*), C-C motif chemokine ligand 11 (*Ccl11*), C-X3-C motif chemokine ligand 1 (*Cx3cl1*), and C-C motif chemokine ligand 5 (*Ccl5*), were increased in eWAT from LPS-treated mice, compared with those of the control mice; whereas pretreatment of α-Man dramatically decreased the expressions of these chemokines (Figure 1D), which was further confirmed by the effects of α-Man on LPS-treated RAW264.7 macrophages (Supplementary Figure 2E). The above results suggested that α-Man reduces pro-inflammatory cytokines and chemokines levels, which, in turn, attenuates inflammatory responses in eWAT from LPS-treated mice.

### α-Man treatment attenuates adipose tissue inflammation through blocking mitogen-activated protein kinases (MAPKs) and nuclear factor-κB (NF-κB) pathways and activating Sirtuin 3 (SIRT3)

F4/80 and CD68 are widely used as macrophage-specific markers. The mRNA levels of *F4/80* and *Cd68* were significantly elevated in eWAT from LPS-treated mice, and pretreatment with α-Man obviously suppressed these gene expressions (Figure 1E), indicating α-Man reduced macrophage content in adipose tissue. Furthermore, α-Man treatment suppressed the expression of M1 macrophage marker *Cd11c* (Figure 1E), and elevated the expression of M2 macrophage markers *Cd206* and *Arginase-1* (*Arg-1*) (Figure 1E), suggesting α-Man promotes a shift towards anti-inflammatory M2 macrophages. The protein levels of macrophage markers further supported the above results (Supplementary Figure 2F).

**Figure 2:**
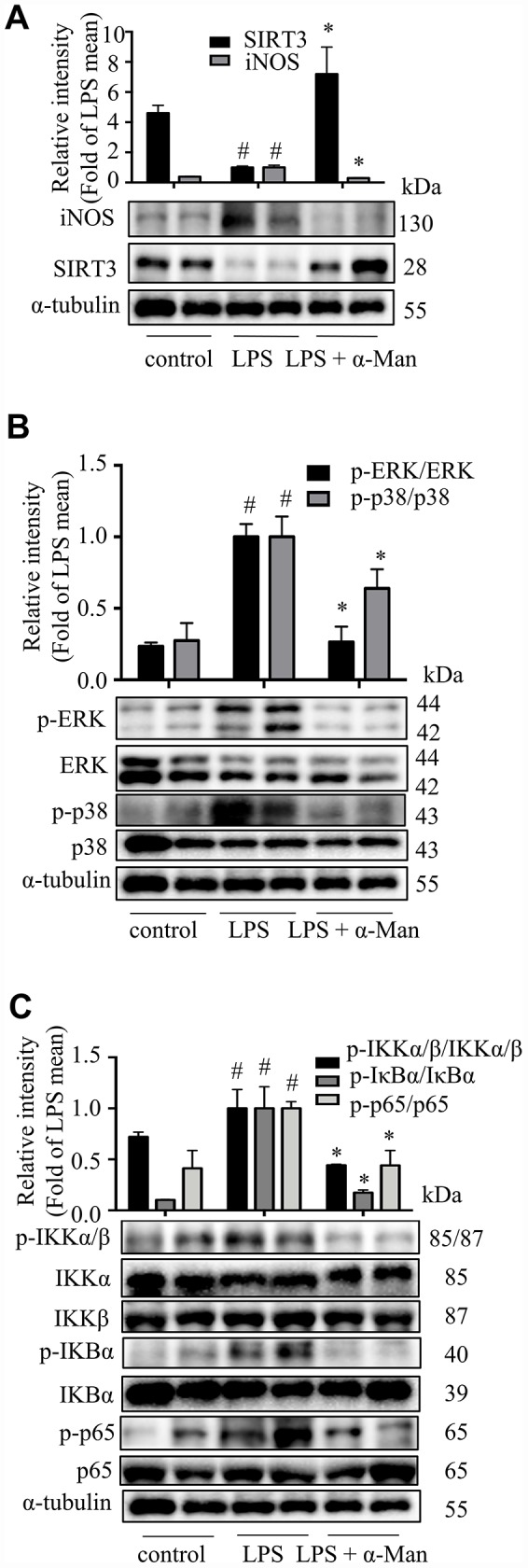
**α-Man blocks MAPKs and NF-κB pathways and activates SIRT3 in eWAT from LPS-treated mice.** (**A**) The expression of iNOS and SIRT3 in eWAT were detected by Western blot analyses. (**B**) The expression of p-ERK, ERK, p-p38 and p38 were detected by Western blot. (**C**) The expression of p-IKKα/β, IKKα, IKKβ, p-IκBα, IκBα, p-p65 and p65 were detected by Western blot. α-Tubulin was used as an internal control. Data are expressed as means ± SD (*n* = 5). ^#^*P* < 0.05 LPS vs. control, ^*^P < 0.05, LPS + α-Man vs. LPS.

**Figure 3:**
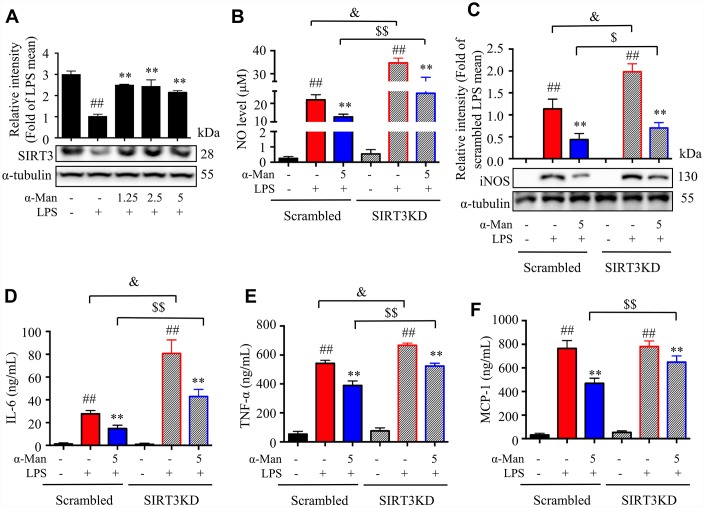
**Effects of α-Man in LPS stimulated SIRT3-knockdown RAW264.7 macrophages.** (**A**) The protein expression of SIRT3 was determined by Western blot in LPS-induced RAW264.7 macrophages. α-Tubulin was used as an internal loading control. Data are normalized to the mean value of LPS group. (**B**) NO production was determined by Griess reagent. (**C**) iNOS abundance was measured by Western blot. α-Tubulin was used as an internal loading control. Data are normalized to the mean value of scrambled LPS group. The levels of IL-6 (**D**), TNF-α (**E**) and MCP-1 (**F**) were determined by ELISA kit. Data are shown as means ± SD (*n* = 5). ^##^*P* < 0.01, LPS vs. DMSO, **P < 0.01, α-Man + LPS vs. LPS, ^&^*P* < 0.05, SIRT3KD LPS vs. scrambled LPS, ^$^*P* < 0.05, ^$$^*P* < 0.01, SIRT3KD α-Man vs. scrambled α-Man.

LPS induces the expression of iNOS (inducible nitric oxide synthase), which mediating the production of nitric oxide (NO), a key inflammatory mediator [20]. Nuclear factor-κB (NF-κB), a nuclear transcription factor, is a regulator of inflammatory processes, which is required for the transcription of numerous cytokines, including TNF-α, IL-1β, and IL-6 [21]. Mitogen-activated protein kinases (MAPKs), including p38 MAPK, extracellular regulated kinase (ERK), and c-Jun NH2-terminal kinase (JNK), are important players in signal transduction of inflammation [22]. The deacetylase Sirtuin 3 (SIRT3) has been reported to activate the MAPKs and NF-κB signaling pathways in an inflammatory model [23]. As shown in Figure 2A, the iNOS expression was increased and SIRT3 expression was decreased in eWAT from LPS-treated mice, and α-Man treatment significantly reversed these changes. The above results were consistent with the effects of α-Man on LPS-induced RAW264.7 macrophages (Supplementary Figure 3A). Moreover, α-Man significantly suppressed the LPS-induced activation of MAPKs and NF-κB pathways in eWAT (Figure 2B, 2C), which were in agreement with the effects of α-Man on LPS-induced RAW264.7 macrophages (Supplementary Figure 3B–3D). Treatment with α-Man abolished the LPS induced IKK activation in macrophages (Supplementary Figure 3E). Furthermore, SIRT3 knockdown partially blocked the anti-inflammatory property of α-Man on RAW264.7 macrophages (Figure 3A–3F). These data demonstrated that α-Man alleviates LPS-induced acute adipose tissue inflammation through inhibiting NF-κB and MAPKs activation and promoting SIRT3 expression.

### α-Man treatment ameliorates adiposity, hyperlipidemia, and insulin resistance in old mice

**Figure 4:**
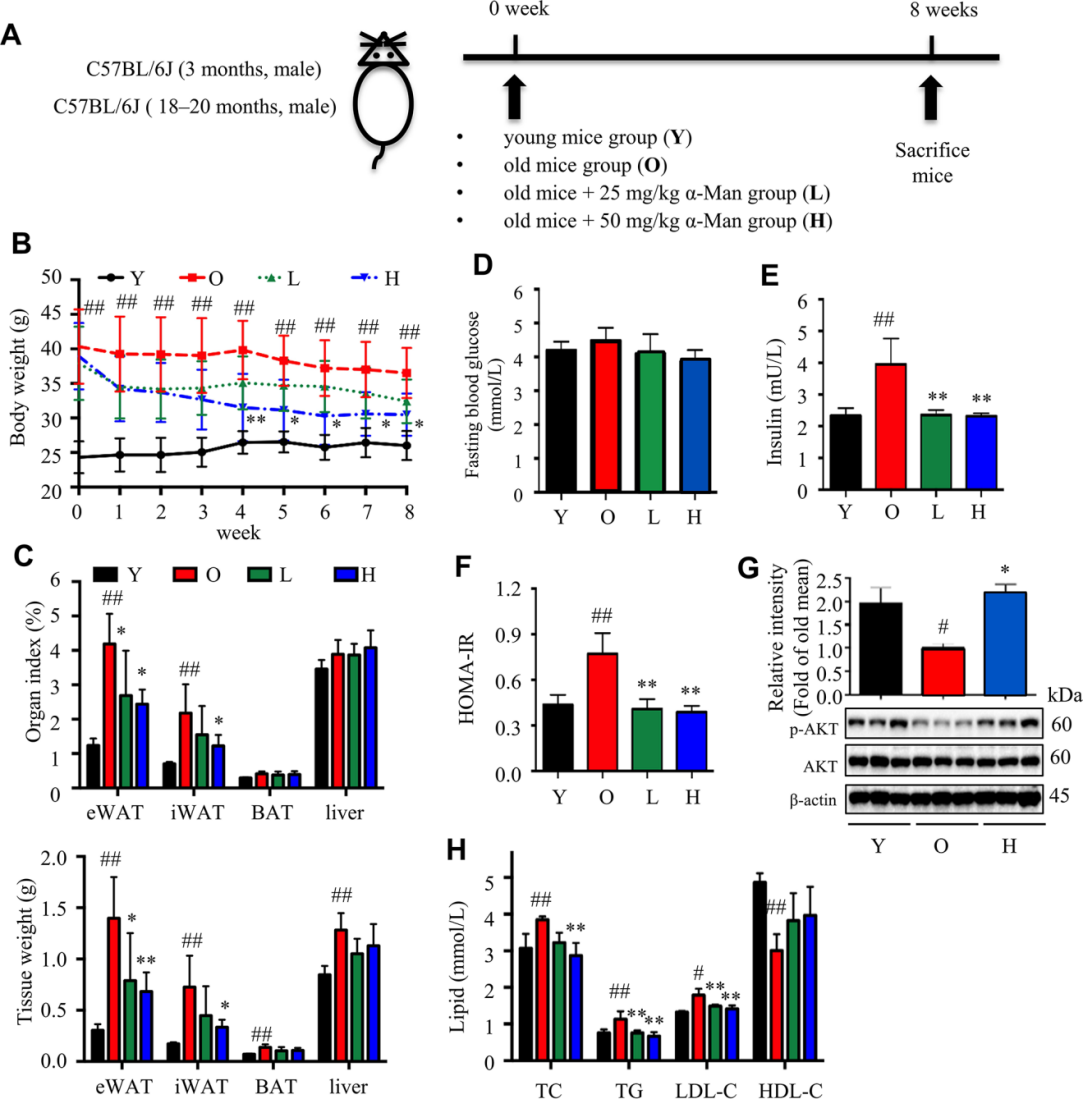
**α-Man ameliorates adiposity, insulin resistance, and hyperlipidemia in old mice.** (**A**) The procedure of α-Man treatment in old mice. (**B**) Effect of α-Man on body weight in old mice. (**C**) The raw tissue weights and organ indexes for each group of mice. Fasting blood glucose (**D**), fasting insulin levels (**E**), and HOMA-IR (**F**) for each group of mice. (**G**) The levels of p-AKT and AKT in eWAT were detected by Western blots. β-Actin was used as internal loading control. (**H**) The levels of serum TC, TG, LDL-C, and HDL-C in serum was detected. Data are expressed as means ± SD (*n* = 5). ^#^*P* < 0.05, ^##^*P* < 0.01, old mice vs. young mice. *P < 0.05, **P < 0.01, α-Man vs. old mice. Y, young mice; O, old mice; L, old mice administrated with 25 mg/kg α-Man; H, old mice administrated with 50 mg/kg α-Man.

Aging is associated with gain of fat mass, hyperlipidemia and insulin resistance. A cohort of young and old mice were recruited to assess the effect of α-Man in ameliorating aging-related metabolic disorders (Figure 4A). α-Man significantly decreased the body weight of old mice since 4 weeks post treatment (Figure 4B and Supplementary Table 1). The increased body weight of old mice might be due to the gain of fat mass, both visceral fat (eWAT) and subcutaneous fat (inguinal WAT, iWAT). Brown adipose tissue (BAT) dissipates energy as heat and is positively correlated with energy expenditure [24]. After eight weeks of treatment the mice were dissected and the weights of each organ were measured. The old mice had increased the weights and indexes of eWAT and iWAT, and the weights of BAT and liver, but not the indexes of BAT or liver, when compared with those of young mice (Figure 4C). Eight weeks of α-Man treatment significantly reduced the weights and indexes of eWAT and iWAT, but didn’t affect those of BAT or liver in old mice (Figure 4C). The glucose tolerance test (GTT) indicated that α-Man treatment didn’t change the glucose disposal rate (Supplementary Figure 4A). After 16 hours fasting, the insulin level was greatly higher, and the blood glucose level remained unchanged in old mice, compared with those of the young mice; and α-Man treatment obviously reduced blood insulin level, but not glucose level (Figure 4D, 4E). Homeostasis model assessment of insulin resistance (HOMA-IR) is a method used to evaluate insulin resistance [25]. The HOMA-IR results indicated α-Man treatment improved insulin sensitivity in old mice (Figure 4F), which was further supported by the increased p-AKT level in eWAT from α-Man-treated old mice (Figure 4G). The serum levels of total cholesterol (TC), triglyceride (TG) and low-density lipoprotein cholesterol (LDL-C) were significantly decreased in the α-Man treated mice compared with those of the old mice (Figure 3H). The serum level of high-density lipoprotein cholesterol (HDL-C) was significantly lower in old mice, and α-Man slightly attenuated the reduction (Figure 4H). Taken together, α-Man treatment ameliorates adiposity, hyperlipidemia, and insulin resistance in old mice.

### α-Man treatment reduces macrophage content and reverses pro-inflammatory macrophage polarization in eWAT from old mice

**Figure 5:**
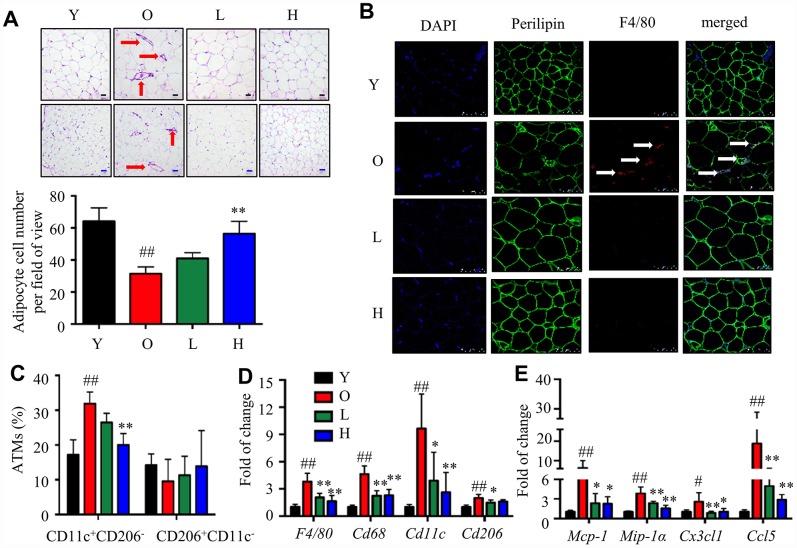
**α-Man mitigates age-related adipose tissue inflammation.** (**A**) H&E staining of eWAT (black scale bar = 50 μm, blue scale bar = 100 μm). (**B**) Whole-mount immunohistochemistry analysis of the nuclei (blue), perilipin (green), and F4/80 (red), scale bar = 75 μm. The CLSs are indicated by arrows (**C**) ATM subtypes were quantified as a percentage of the total ATMs population using flow cytometry. (**D**) qRT-PCR analyses for macrophage markers in eWAT, including *F4/80*, *Cd68*, *Cd11c*, and *Cd206*. (**E**) qRT-PCR analyses for chemokines in eWAT, including *MCP-1*, *MIP-1α*, *Cx3cl1*, and *Ccl5*. Data are normalized to the mean value of old group. Data are expressed as means ± SD (*n* = 5). ^#^*P* < 0.05, ^##^*P* < 0.01, old mice vs. young mice, *P < 0.05, **P < 0.01, α-Man vs. old mice. Y, young mice; O, old mice; L, old mice administrated with 25 mg/kg α-Man; H, old mice administrated with 50 mg/kg α-Man.

To test whether α-Man reduced inflammation in eWAT from old mice, ATM content and polarization were analyzed. Crown-like structures (CLS), composed of macrophages surrounding dead or dying adipocytes, are a histologic hallmark of adipose tissue inflammation [26]. The H&E staining of eWAT from old mice exhibited bigger adipocytes and more CLSs when compared with the young mice; while α-Man treatment significantly reduced the adipocyte size and the amount of CLSs (red arrows) in eWAT (Figure 5A). The immunohistochemical staining of eWAT from old mice showed more F4/80^+^ macrophages, when compared with the young mice; while α-Man treatment significantly reduced the adipocyte size and the amount of F4/80^+^ macrophages (white arrows) in eWAT (Figure 5B, Supplementary Figure 4B). The above observation indicated α-Man treatment reduced the amount of dead adipocytes and macrophage content in eWAT from old mice. Furthermore, three major subsets of ATMs in eWAT were analyzed by flow cytometry that parallel prior studies: M1 ATMs (CD11c^+^CD206^-^), M2 ATMs (CD11c^-^CD206^+^), and double negative (DN) ATMs (CD206^-^CD11c^-^) [8]. M1 macrophages were significantly increased, and M2 and DN macrophages were slightly decreased with age; and α-Man treatment significantly decreased the content of M1 macrophages, but didn’t change the contents of M2 or DN macrophages (Figure 5C, Supplementary Figure 4C and 4D). With age, the ratio of pro-inflammatory M1 ATMs to anti-inflammatory M2 ATMs was slightly increased, demonstrating pro-inflammatory polarization of ATMs (Supplementary Figure 4E). Interestingly, α-Man treatment slightly reversed the M1/M2 ratio in eWAT (Supplementary Figure 4E).

Next, the gene expressions of macrophage markers and chemokines were examined. The expression of *F4/80* and *Cd68* in eWAT was increased with age, and significantly reduced in α-Man treated old mice (Figure 5D). The expression of *Cd11c* and *Cd206* was increased during aging; remarkably, α-Man down-regulated the expression of *Cd11c*, and slightly suppressed the expression of *Cd206* (Figure 5D). The protein level of macrophage markers further supported the above results (Supplementary Figure 4F). The mRNA levels of chemokines including *Mcp-1*, *Mip-1α*, *Cx3cl1*, and *Ccl5* were significantly elevated in eWAT from the old mice when compared with those of the young mice; whereas α-Man treatment greatly suppressed the expression of these chemokines (Figure 5E), suggesting that α-Man prevents aging-related macrophage infiltration into adipose tissue. It seemed that the reduction of ATMs content in α-Man treated old mice reflected decreased chemotaxis of macrophages. To test this hypothesis, the migration capacity of RAW264.7 macrophages towards CM from 3T3-L1 adipocytes was evaluated using a transwell chemotaxis assay. RAW264.7 macrophages were seeded in the upper chamber of the Transwell plate and induced to migrate in the presence of DMEM or adipocyte CM in the lower chamber (Supplementary Figure 5A). Pretreatment of α-Man to the macrophages prevented the migration of macrophages towards adipocyte CM (Supplementary Figure 5B, 5C). These data indicated that α-Man reduces macrophage content and promotes ATMs polarization towards an anti-inflammatory M2 state in eWAT from old mice.

### α-Man ameliorates age-related adipose tissue inflammation through NF-κB and MAPKs pathways

**Figure 6:**
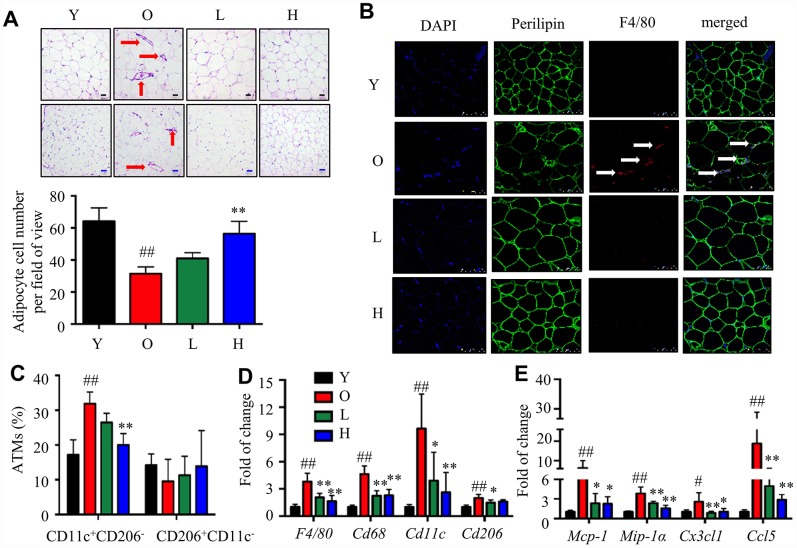
**α-Man mitigates age-related adipose tissue inflammation through NF-κB and MAPKs pathways.** (**A**) Relative mRNA levels of *iNos*, *Il-1β* and *Tnf-α* in eWAT were analyzed by qRT-PCR. (**B**) The protein levels of iNOS, COX-2 and SIRT3 in eWAT were detected by Western blot analyses and quantified using Image J. (**C**) The expression of p-IKKα/β, IKKα, IKKβ, p-IκBα, IκBα, p-p65 and p65 was detected by Western blot. (**D**) The expression of p-ERK, ERK, p-p38, p38, p-JNK and JNK were detected by Western blot. α-Tubulin was used as an internal control. Data are normalized to the mean value of old group. Data are expressed as means ± SD (*n* = 5). ^#^*P* < 0.05, ^##^*P* < 0.01, old mice vs. young mice, ^*^P < 0.05, ^**^P < 0.01, α-Man vs. old mice. Y, young mice; O, old mice; L, old mice administrated with 25 mg/kg α-Man; H, old mice administrated with 50 mg/kg α-Man.

The mRNA levels of *iNos*, *Tnf-α*, *Il-1β* in eWAT were increased with age, and significantly reduced in eWAT from α-Man treated old mice (Figure 6A), indicating α-Man treatment reduced adipose tissue inflammation in old mice. As shown in Figure 6B, aging was accompanied with increased iNOS and COX-2 levels, and decreased SIRT3 level in eWAT, and α-Man treatment reversed the changes. Compared to the young mice, the phosphorylation of IKKα/β, IκBα and p65 was increased remarkably in eWAT from old mice, while significantly decreased after α-Man treatment (Figure 6C). Regarding the MAPKs pathway, the phosphorylated ERK and p38 were elevated in eWAT from old mice when compared with the young mice, and α-Man significantly suppressed the phosphorylation of ERK and p38 (Figure 6D). These data suggested that α-Man ameliorates aging-related inflammatory responses in adipose tissue, at least in part, through inhibiting the activation of NF-κB and MAPKs signaling pathways.

### α-Man ameliorates aging-related liver injury by suppressing miR-155 secretion from macrophages

**Figure 7:**
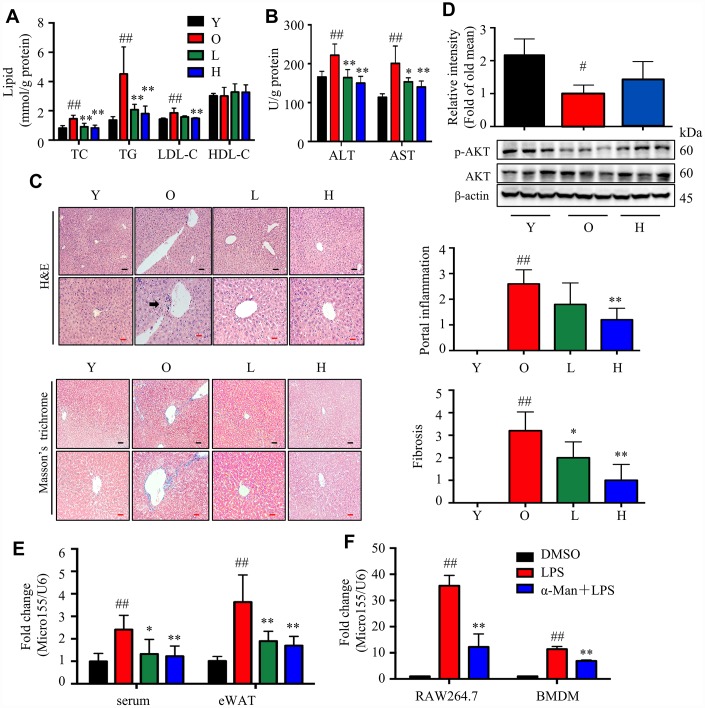
**α-Man alleviates liver injury in old mice by inhibiting *miR155* expression.** (**A**) The levels of TC, TG, LDL-C and HDL-C in livers (*n* = 5). (**B**) The levels of ALT and AST in livers (*n* = 5). (**C**) H&E staining and Masson’s trichrome staining of liver tissues, and histopathological scores of individual livers on portal inflammation and fibrosis. (*n* = 5). Black scale bar = 100 μm, red scale bar = 50 μm. Black arrows indicate sites of portal inflammation. 0 = no significant change, 1 = minimal, 2 = mild, 3 = moderate, and 4 = severe pathology. (**D**) The levels of p-AKT and AKT were detected by Western blot (*n* = 5). β-Actin was used as an internal loading control. (**E**) The expression level of *miR-155* in the serum and eWAT from mice (*n* = 5). Data were normalized to level of *U6* snRNA. Data are expressed as means ± SD. ^#^*P* < 0.05, ^##^*P* < 0.01, old mice vs. young mice, *P < 0.05, **P < 0.01, α-Man vs. old mice. Y, young mice; O, old mice; L, old mice administrated with 25 mg/kg α-Man; H, old mice administrated with 50 mg/kg α-Man. (**F**) The expression level of *miR-155* in LPS stimulated RAW264.7 macrophages and BMDMs (*n* = 6). Data were normalized to level of *U6* snRNA. Data are expressed as means ± SD. ^##^*P* < 0.01, LPS vs. vehicle, **P < 0.01, α-Man + LPS vs. LPS.

Adipose tissue inflammation is positively correlated with liver injury [27]. Next, we evaluated whether α-Man improved liver injury in old mice. The levels of TC, TG and LDL-C were significantly decreased, but not HDL-C, in liver from the α-Man treated mice, compared with those of the old mice (Figure 7A). Furthermore, the alanine aminotransferase (ALT) and aspartate aminotransferase (AST) levels were significantly reduced by α-Man treatment compared with those of the old mice (Figure 7B). H&E and Masson’s trichrome straining results showed severe liver injury in old mice, which was remarkably rescued by α-Man treatment (Figure 7C). Compared to the young mice, the p-AKT was significantly decreased in old mice, and α-Man reversed this decline (Figure 7D).

*microRNA-155-5p* (*miR-155*) is induced during inflammatory response in macrophages [28], which is a key mediator of liver steatosis and fibrosis in alcohol-induced mice [29]. Herein, the level of *miR-155* in serum and eWAT from old mice was higher than that of the young mice, which was reduced in α-Man treated old mice (Figure 7E). Consistently, α-Man treatment decreased the *miR-155* expression in LPS stimulated RAW264.7 macrophages and bone marrow derived macrophages (BMDMs) (Figure 7F), but didn’t show obvious cytotoxicity on both cell lines up to 5 μM, with or without LPS (Supplementary Figure 6). Thus, α-Man protects old mice against liver injury, mediating through the suppression of *miR-155* secretion from macrophages.

## DISCUSSION

Besides energy storing and providing function, more and more evidence has suggested that adipose tissue serves as a major endocrine organ, secreting free fatty acids, adipokines and chemokines to modulate energy and glucose homeostasis [30]. The accumulation of visceral fat is a common feature of aging, which is considered to be the most detrimental factor on aging-associated metabolic syndrome in humans [31]. Obesity is positively correlated with increased pro-inflammatory cytokines and enhanced adipose tissue inflammation [7]. Thus, therapy targeting allievation of adipose tissue inflammation might be effective in preventing and treating aging-related metabolic diseases. In the current study, α-Man was discovered to ameliorate aging-related adiposity, hyperlipidemia, insulin resistance and liver injury by alleviating adipose tissue inflammation, possibly through inhibiting NF-κB and MAPKs signaling pathways.

**Figure 8:**
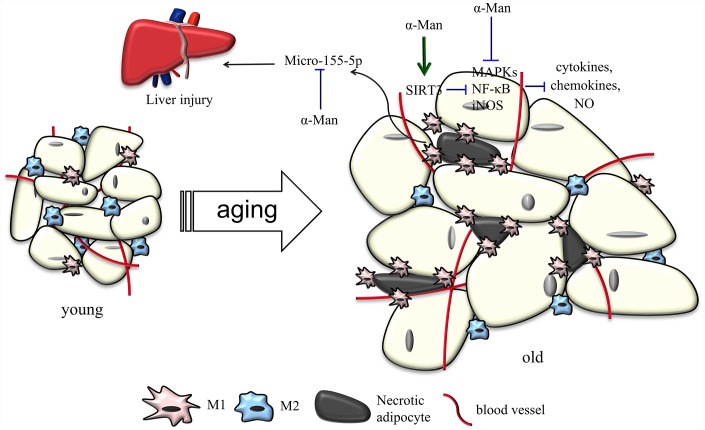
**Schematic models of molecular targets of α-Man in attenuating visceral adipose tissue inflammation.**

Natural products are important source of therapeutic agents to ameliorate inflammatory responses in adipose tissue and treat aging-related metabolic disorders. Dietary curcumin supplementation reduces macrophage infiltration and increases the ratio of M2/M1 macrophages in WAT from HFD-fed mice [32]. Corosolic acid, called “phyto-insulin”, was reported to improve adipose tissue inflammation and insulin resistance in HFD-fed mice [33]. Resveratrol, a sirtuin1 (SIRT1) activator, inhibits NF-κB activation and improves insulin sensitivity in eWAT from high-fat, high-sugar fed rhesus monkeys [34]. 1,3,6,7-Tetrahydroxy-8-prenylxanthone was found to alleviate adipose tissue inflammation through modulating macrophage phenotype and chemotaxis in eWAT from LPS-treated mice [17]. α-Man has been widely reported with anti-obesity and anti-inflammatory properties [15, 16, 35, 36]. α-Man inhibits p65 acetylation and reduces COX-2 and iNOS gene expression *via* activating SIRT1 in LPS-treated human monocytes [35]. α-Man suppresses adipocyte differentiation and reduces adiposity in HFD fed mice [15, 16]. The current study provided the first evidence that α-Man reverses inflammatory responses in adipose tissue from LPS-treated or aging mice, which uncovers the beneficial effects of α-Man on aging-related hyperlipidemia, insulin resistance and liver injury (Figure 8). Thus, α-Man might represent a new class of scaffold for treatment of metabolic diseases in the elderly. Nevertheless, we cannot exclude the possibility that α-Man might improve aging-associated metabolic disorders through other tissues, such as liver and skeletal muscle.

Recruitment and activation of macrophages in the expanding adipose tissues promote low-grade inflammation during aging. The majority of adipose tissue-derived cytokines originate from infiltrating macrophages, which ultimately increase circulating pro-inflammatory cytokines to develop low-grade chronic inflammatory state [37]. Herein, α-Man was found to decrease pro-inflammatory cytokines in LPS-treated RAW264.9 macrophages and eWAT from LPS-treated or aging mice. Unbalanced production of pro-inflammatory and anti-inflammatory cytokines in WAT contributes to the development of metabolic syndrome, such as insulin resistance and type 2 diabetes [38]. Previous report has suggested that aging is associated with unchanged M1 ATMs, and a concomitant decrease in M2 ATMs [8]. While, our previous study indicated an increase in total ATMs, as well as M1 and M2 ATMs, during aging [39]. The current data showed aging is associated with increased M1 ATMs and unchanged M2 ATMs. The different ATM profiles in eWAT from aged mice might be due to the age of mice and the mothed for quantitating ATMs. The aged mice were 18–20 months old in current study, while 13–16 and 18–22 months old in our previous study and Lumeng’s study, respectively [8, 39]. To analyze the ATM content in eWAT, the similar SVF isolation and flow cytometry methods were used in our previous and current studies [39]. In Lumeng’s study, CD11b^+^ ATMs were firstly separated from the total SVF using the MACS Microbeads technology, which were then used for flow cytometry analysis [8]. Intriguingly, the ratio of M1/M2 ATMs was found to be increased with age in the current study, which is consistent with previous reports. It further supported that aging is accompanied with macrophage polarization towards pro-inflammatory phenotype. Interestingly, α-Man treatment resulted in an anti-inflammatory phenotypic shift of macrophages in both LPS-treated and aging mice. The current study provides a novel mechanism for α-Man in treating metabolic disorders.

Chemokines and cytokines are involved in a variety of physiologic and pathologic processes. MCP-1 and MIP-1α are the key chemokines for macrophage recruitment into adipose tissue [37]. Currently, the levels of MCP-1 and MIP-1α were up-regulated in eWAT from LPS stimulated or old mice, and α-Man reversed the increase of chemokines. In mice and humans, MCP-1 production is increased in plasma and adipose tissue in both diet-induced and genetically-induced obesity, and MCP-1 promotes the recruitment of monocytes into the expanding adipose tissues [40]. In adipose tissue, both adipocytes and the infiltrated immune cells are responsible for the secretion of MCP-1. MIP-1α is robustly up-regulated in WAT from obese mice, and the infiltrated ATMs secrete MIP-1α in inflamed fat [41]. We also found that the levels of Cxc110, ccl11, cx3cl1 and Ccl5 were up-regulated in eWAT from LPS stimulated mice, and the levels of cx3cl1 and Ccl5 were increased in eWAT from old mice, which were reversed by α-Man treatment; and α-Man directly inhibited the migration of macrophages towards adipocytes. Cxcl10 is up-regulated in ATMs from *ob/ob* mice [42], Cx3cl1, Ccl11 and Ccl5 are increased in adipose tissue from obese mice [43], and the expression of these chemokines is mainly regulated by NF-κB signaling pathway [44]. These evidence suggested that the reduced ATMs content in α-Man-treated mice might be through blocking the infiltration of monocytes from circulation.

SIRT3 expression is down-regulated with age, and its deficiency is accompanied with mitochondrial protein hyperacetylation [45], resulting in aging-associated diabetes, cancer, and inflammation [46]. Consistently, our data showed that SIRT3 level is decreased in LPS-treated macrophages, and in adipose tissue from LPS-treated or aging mice, which supported that SIRT3 represents an anti-inflammatory target. SIRT3 attenuates palmitate-induced inflammation in proximal tubular cells through regulating mitochondrial oxidative capacity and antioxidant gene expression [23]. Mice lacking SIRT3 develop several aging related diseases, which could be considered as a model of accelerated aging [47]. Overexpression of SIRT3 in HEK293 cells induced hypoacetylation and affected the intracellular localizations and protein stabilities of their interacting partners, which are critically involved in the anti-aging and metabolic regulatory activities [48]. Interestingly, α-Man increases SIRT3 expression level, which might mediates the protective role of α-Man against aging-associated insulin resistance, hyperlipidemia and liver injury. Further studies are needed to figure out whether α-Man could directly bind to SIRT3 and how α-Man regulates SIRT3 expression.

miRNAs are small non-coding RNAs that modulate the target genes expression involved in diverse biological processes. miRNAs could be a link among inflamm-aging, obesity and diabetes [49–51]. miR-155 is a common target of a broad range of inflammatory mediators, and is induced during inflammatory response in macrophages [28]. α-Man was found to suppress the level of miR-155 in LPS-induced RAW274.7 macrophages and BMDMs. ATMs in obese subjects secret miR-155-containing exosomes, which causes glucose intolerance and insulin resistance in adipocytes, myocytes, and primary hepatocytes [52]. TNF-α increases miR-155 expression in adipocytes, which results in obesity progression in female mice by limiting BAT differentiation [53]. Silencing miR-155 causes depression of CCAAT/enhancer binding protein β and down-regulation of granulocyte colony-stimulating factor in LPS-treated mice [54], and attenuates liver steatohepatitis and fibrosis in alcohol-induced mice [29]. miR-155 is increased in serum/plasma in alcoholic and inflammatory liver injury [55]. miR-155 targets NF-κB and MAPKs pathway, which are the inflammatory response crossroad among macrophage, adipocyte and aging [56, 57]. α-Man decreased miR-155 expression in serum and eWAT from old mice, which might benefit the improvement of liver injury in α-Man-treated old mice. It will be interested to further explore the effects of miR-155 on the dynamics of intracellular cAMP production, as it has previously been linked to granular priming and exocytosis. Thus, the protective effect of α-Man against liver injury in old mice might be associated with the suppression of both pro-inflammatory cytokines and miR-155.

α-Man alleviated age-associated insulin resistance, hyperlipidemia and liver injury, which might also be due to its effect on adiposity. At the current stage, we couldn’t classify which the major contributor is in aging-related adipose tissue inflammation, macrophage or adipocyte. In addition to macrophages, numerous other types of immune cell populate the adipose tissue and affect its function. The current study didn’t evaluate the content of lymphocytes and monocytes in WAT from α-Man-treated mice. Additionally, the current study only focused on visceral adipose, subcutaneous adipose may also contribute to aging-associated adipose tissue inflammation. Sex differences were not determined in the current study. Most importantly, the direct target responsible for the suppressing effect of α-Man on cytokines and chemokines was still unclear. In the future, the primary adipocytes and BMDMs will be isolated from aged mice, to generate a co-culture system. α-Man will be added in the culture medium of mature adipocytes or BMDMs alone, to figure out which is the major contributor mediating the effect of α-Man in alleviating adipose tissue inflammation during aging. Using biotin-labelled α-Man, the direct target of α-Man will be identified in future study. Furthermore, the target protein deficiency animal model will be recruited to confirm the effect of α-Man. T lymphocytes and monocytes have been discovered to participate in aging-associated adipose tissue inflammation. Further studies will be carried out to evaluate the effect of α-Man on these immune cells in adipose tissue.

## CONCLUSIONS

In summary, the current study revealed that α-Man ameliorates inflammatory responses in eWAT from LPS-induced or aging mice, possibly through inhibiting NF-κB and MAPKs signaling pathways and increasing SIRT3 expression. Furthermore, α-Man reduces macrophage content and shifts macrophage towards anti-inflammatory state in adipose tissue to alleviate adipose tissue inflammation, which, in turn, ameliorates aging-related adiposity, hyperlipidemia, insulin resistance and liver injury. α-Man improves adipose tissue inflammation, which might be developed as a candidate for treatment of aging-related metabolic disorders.

## MATERIALS AND METHODS

### Ethic

All procedures involved in the animal experiments were carried out in accordance with the approved guidelines and regulations by the Animal Ethical and Welfare Committee of University of Macau (No. ICMS-AEC-2014-06). Animal studies and primary cultures are reported in compliance with the ARRIVE guidelines. Experimental protocols and design are reported in compliance with the guidelines.

Male C57BL/6J were obtained from the Faculty of Health Science, University of Macau (Macau, China). The mice were housed in plastic cages and fed with a regular chow diet (18% protein, 4.5% fat, and 58% carbohydrate, Guangdong Medical Lab Animal Center, Guangzhou, Guangdong, China) and water *ad libitum* under standard conditions (specific-pathogen-free) with air filtration (22 ± 2 °C, 12-h light/12-h dark). The authors declare that the data supporting the findings of this study are available within the article.

### LPS-induced acute inflammation mice

Acute inflammation mice model was generated by a single injection of LPS (*Escherichia coli*, serotype 0111: B4, Sigma-Aldrich, St. Louis, MO, USA) as reported previously [17]. The male C57BL/6J mice (8–10 weeks old) were randomly divided into three groups according to body weight (*n* = 5 in each group). The mice in α-Man group were intraperitoneally injected with α-Man (10 mg**/**kg/d, dissolved in polyethylene glycol 400 (PEG 400): distilled water = 6:4, v/v), and the mice in LPS and control group were intraperitoneally injected with vehicle solution (10 mL/kg, PEG 400:distilled water = 6:4, v/v) once a day for consecutive 5 days. On the sixth day, the mice in control group were intraperitoneally injected with PBS, and the mice in LPS and α-Man group were intraperitoneally injected with LPS (4 mg**/**kg, dissolved in PBS). Four hours after LPS injection, the blood samples were collected from mice under inhalational anesthesia with isoflurane. Then the mice were euthanatized by carbon dioxide, and the eWATs were dissected and stored at -80 °C.

### Adipose tissue inflammation in old mice

{Che, 2014 #783}A cohort of young (3 months old) and old (18–20 months old) C57BL/6J male mice were recruited. The old mice were randomly divided into three groups according to body weight (*n* = 5 in each group). The mice in α-Man-25 (low dosage, L) and α-Man-50 (high dosage, H) groups were orally administrated with 25 and 50 mg**/**kg/d of α-Man (dissolved in PEG 400 solution) once a day for 8 weeks, respectively; and the old (O) and young (Y) control group of mice were orally administrated with the same volume of vehicle solution (10 mL**/**kg, PEG 400:distilled water = 6:4, v/v). At the end of experiment, the blood samples were collected from mice under inhalational anesthesia with isoflurane. The mice were euthanatized by carbon dioxide, and the eWAT, iWAT, BAT and liver were dissected. One part of eWAT was quickly rinsed in Krebs-Ringer bicarbonate (KRB) buffer for isolation of SVF, another part was fixed in 4% paraformaldehyde for histochemical and immunohistochemical analyses, and the remaining eWATs were stored at -80 °C for subsequent Western blotting and qRT-PCR analyses.

### Cell culture

Mouse derived RAW264.7 macrophages (passages 5–15, American Type Culture Collection, ATCC, Manassas, VA) and 3T3-L1 preadipocytes (passages 7–10, ATCC) were cultured and differentiated as previously reported [17]. Cells were cultured under a humidified 5% (v/v) CO_2_ atmosphere at 37 °C. Briefly, 2-day post-confluent 3T3-L1 preadipocytes were stimulated for 72 hours with 0.5 mM isobutylmethylxanthine (Sigma-Aldrich), 1 μM dexamethasone (Sigma-Aldrich), and 5 μg/mL insulin (Sigma-Aldrich) in DMEM supplemented with 10% FBS. Cells were subsequently treated with DMEM supplemented with 10% FBS and 5 μg/mL insulin for 5 days. The fully differentiated 3T3-L1 cells were confirmed by microscopic observation and Oil-Red O staining as described previously [58, 59]. All treatments were performed on day 8 post differentiation. RAW264.7 macrophages were seeded and cultured in DMEM with 10% FBS for 24 hours. RAW264.7 macrophages were pre-treated with α-Man (5 μM) for 1 hour and then stimulated with LPS (1 μg**/**mL) for 6 hours. Subsequently, the cells were harvested for the following studies.

### Silencing of SIRT3 in macrophages

The shRNA targeting SIRT3 (mouse, sc-61556-SH), control shRNA plasmid-A (mouse, sc-108060), and shRNA transfection reagent (mouse, sc-108061) were purchased from Santa Cruz Biotechnology (Santa Cruz, CA, USA). RAW264.7 cells at 50% confluency were transfected with 4 μg shRNA for 6 hours according to the manufacturer's protocol. Cells were switched to fresh medium and incubated for an additional 42 hours. Then, cells were selected with 10 μg**/**mL puromycin (Gibco) for 14 days. Thereafter, cells were pooled together for further experiments.

### SVF isolation and ATMs analysis with flow cytometry

The SVF from eWAT was isolated as previously described [60, 61]. eWAT from each mouse was minced with scissors in KRB buffer. After centrifuged at 400 g for 5 min to remove blood cells, fat tissue was incubated in KRB buffer with 1 mg/mL type I collagenase (Worthington Biochemical Corporation, NJ, USA) at 37 °C for 40 min with shaking. The tissue slurry was filtered through a 100 μm filter to remove the undigested tissue. After centrifuged at 400 g for 5 min, SVF pellet was collected in the bottom. The SVF pellet was resuspended in PBS, and around 1 × 10^6^ cells were blocked with anti-CD16/32 antibody (BD Bioscience, San Jose, CA, USA) for 30 min on ice. Then the cells were incubated with antibodies against F4/80 (eBioscience, San Diego, CA, USA), CD11c (BD Bioscience) and CD206 (BioLegend, San Diego, CA, USA) for 30 min on ice followed by 2 washes in PBS. Next, the cells were treated with Red Blood Cell Lysis Buffer (BD Bioscience) for 10 min on ice followed by PBS washing. The cells were resuspended in PBS, and the data were collected using an Accuri™ C6 flow cytometer (BD Bioscience) and analyzed by FlowJo software (Tree Star, Ashland, OR, USA).

### Measurement of metabolic parameters

HDL-C, LDL-C, TG, and TC in serum and liver, and AST and ALT in liver were detected by using commercial kits (Nanjing Jiancheng Bioengineering Institute, Nanjing, Jiangsu, China). The glucose tolerance tests (GTT) were performed at six weeks post α-Man treatment, as described previously [62]. After 16 hours fasting, the tail blood glucose was measured using OneTouch Ultra blood glucose meter and LifeScan test strips. Then, the mice were intraperitoneally injected with glucose solution (Sigma-Aldrich, St Louis, MO, USA) at a dose of 2.0 g**/**kg body weight. The tail blood glucose was measured at 15, 30, 60, 90 and 120 min after injections. And seven weeks after the first administration, the serum insulin was determined by commercial enzyme-linked immunosorbent assay (ELISA) kits (Mercodia AB, Uppsala, Sweden) after 16 hours fasting. A homeostasis model assessment of insulin resistance (HOMA-IR) was used to evaluate insulin resistance, calculated with the following formula: fasting insulin (mIU**/**L) × fasting blood glucose (mmol**/**L) / 22.5.

### Differentiation of bone marrow-derived macrophages

BMDMs from 6 to 8 weeks old C57BL/6J male mice were prepared as previously described [63]. Briefly, BMDMs were differentiated in RPMI medium 1640 supplemented with 10% FBS and 20% M-CSF-conditioned medium from L929 cells (Stem Cell Bank, Chinese Academy of Sciences) for 7 days. After differentiation, the BMDMs were treatment with α-Man (5 μM) for 1 hour, stimulated with LPS for 6 hours, and then collected for qRT-PCR.

### Histochemical and immunohistochemical analyses

Whole-mount immunohistochemical analysis of eWAT and liver was performed as previously described [39]. After fixation in 4% paraformaldehyde, the tissue samples were embedded in paraffin. 5 μm sections were cut for histological and immunohistochemical analysis. For histological evaluation, sections were deparaffinized and rehydrated followed by haematoxylin and eosin (H&E) staining and Masson’s trichrome staining. To evaluate the degree of liver injury, an injury grading score (Grade 0–4) based on severity of portal inflammation and fibrosis in the liver parenchyma were carried out as previously reported [64, 65]. For immunohistochemical staining, the eWAT sections were deparaffinized and stained with primary antibodies against perilipin (1:100, Cell Signaling Technology, MA, USA) and F4/80 (1:400). After incubation with fluorescently labeled secondary antibodies (Thermo Fisher Scientific) and 4'6-diamidino-2-phenylindole (DAPI) staining, the samples were examined by confocal microscope (Leica, Germany).

### Chemotaxis migration assay

Chemotaxis of RAW264.7 macrophages was performed by using Transwell inserts with a pore size of 8 μm membrane (Corning Incorporated, Corning, NY, USA) as previously described [66, 67]. Briefly, mature 3T3-L1 adipocytes were used for preparation of CM in DMEM (serum free, 0.2% BSA). RAW264.7 macrophages were placed in the upper chamber at a density of 5 × 10^4^ cells per well, and pre-incubated with or without α-Man (5 μM) for 4 hours in serum-free DMEM supplemented with 0.2% BSA. The inserts with macrophages were put into the lower chamber with 0.5 mL adipocyte CM. After migration for 4 hours at 37 °C, the macrophages on the bottom side of the upper chamber membrane were fixed in 4% paraformaldehyde and stain with DAPI.

### Determination of NO levels

The levels of NO were determined by using Griess reagent (Sigma-Aldrich) as previously reported [17].

### Determination of cytokines

The cell culture medium and blood from mice were centrifuged at 4,000 g for 10 min at 4 °C, and the supernatant and serum were collected, respectively. TNF-α, IL-6 and MCP-1 in cell medium, mice serum and eWAT lysates were determined by using commercial ELISA kits (Neobiosciences, Shenzhen, China), according to the manufacturer’s instructions. The cytokine levels in eWAT were further normalized to protein content or tissue weight.

### Nuclear and cytoplasmic protein extraction

The nuclear and cytoplasmic protein was collected with the Nuclear and Cytoplasmic Protein Extraction Kit (Beyotime Biotechnology company, Shanghai, China), according to the manufacture’s instruction.

### Immunoprecipitation and enzyme activity of the endogenous IKK complex

The IKK activity was measured as previously reported [68]. RAW264.7 macrophages were seeded in 10 cm dishes. After 24 hours, the macrophages were pretreated with α-Man (5 μM) or vehicle (DMSO) for 4 hours and then stimulated with LPS (1 μg/mL) for 15 min. Cells were washed with PBS and lysed at 4°C with lysis buffer containing cocktail and PMSF. Afterwards, cell debris was removed by centrifugation for 30 min at 21,000 g. The supernatants were swirled with anti-IKKγ antibody (Santa Cruz) for 1.5 hours at 4°C. The protein A/G PLUS-agarose beads suspension was then added and the supernatants were rotated for overnight at 4°C. Afterwards, the beads were washed twice with lysis buffer, three more times with 1 mL buffer (25 mM HEPES pH 7.4, 2 mM MgCl_2_ and 63 μM ATP). The enzymatic activity of the immunoprecipitated IKK was determined with CycLex IKK Kinase Assay (MBL International, Woburn, MA, USA) according to the manufacturer’s instructions.

### qRT-PCR analysis

Total RNA from cells or tissue was isolated using TRIzol Reagent and was reverse transcribed using the SuperScript III First-Strand Synthesis System (Thermo Fisher Scientific, Grand Island, NY, USA). cDNA samples were amplified by using SYBR green PCR Master Mix (Thermo Fisher Scientific) with gene specific primers (Supplementary Information Table 2). *18S* RNA was used as an internal control. RNAs from serum were isolated with mirVana PARIS Kit (Thermo Fisher Scientific) in conjunction with the synthetic spike-in control (cel-miR-39 mimic, QIAGEN, Hilden, Germany). Reverse transcription reactions for miRNA are performed using the TaqMan miRNA Reverse Transcription Kit (Thermo Fisher Scientific) and miRNA-specific stem-loop primers (Thermo Fisher Scientific). The RT products of serum were pre-amplified (Thermo Fisher Scientific) prior to the real-time PCR step to potentially enhance sensitivity. Real-time PCR reactions for miRNA are performed using the TaqMan 2X Universal PCR Master Mix II (Thermo Fisher Scientific) and TaqMan miRNA-specific probe mix (Thermo Fisher Scientific). Relative quantities of miRNAs were calculated by using the 2^-ΔΔCt^ method with *U6* snRNA (Thermo Fisher Scientific) as the endogenous control. The assays were listed in Supplementary Information Table 3.

### Western blot analysis

Western blot analysis was performed as described previously [17]. In brief, protein concentration was measured by using a BCA protein assay kit. Equal amount of proteins (15–30 μg) were separated by SDS-PAGE and transferred to polyvinylidene fluoride membranes. After blocked with 5% non-fatted milk for 1 hour, the blots were probed with specific primary antibodies (Supplementary Table 4) overnight at 4 °C, and then incubated with horseradish peroxidase conjugated secondary antibody for 1 hour at room temperature. The immune-blotting signals were detected by using SuperSignal West Femto Maximum Sensitivity Substrate (Thermo Scientific) under visualization in ChemiDoc™ MP Imaging System (Bio-Rad).

### Statistical analysis

Data were analyzed using GraphPad Prism 6.0 software. All experimental data were presented as mean ± SD. Statistical analysis of differences between two treatment groups was performed using independent-samples’ t-test. For multiple comparisons, one-way or two-way ANOVA with Bonferroni’s correction was applied. The one-way ANOVA was used for statistical comparison, and p-values less than 0.05 were considered statistically significant.

### Materials

DMEM, RPMI medium 1640, FBS and puromycin were purchased from Life Technologies/Gibco Laboratories (Grand Island, NY, USA). Bovine calf serum was obtained from HyClone (Logan, UT, USA). LPS (*Escherichia coli*, serotype 0111: B4), DAPI, isobutylmethylxanthine, dexamethasone, insulin, and Griess reagent were purchased from Sigma-Aldrich (St. Louis, MO, USA). ELISA kits for MCP-1, IL-6 and TNF-α were obtained from Neobioscience (Shenzhen, China). Antibodies against iNOS, COX-2, SIRT3, p65, p-p65, p-IKK-α/β, IKK-α, IKK-β, p-IκB-α, IκB-α, p-p38, p38, p-ERK, and ERK were purchased from Cell Signaling Technologies (Beverly, MA, USA). Antibodies against p-JNK, JNK, α-tubulin, and GAPDH and Histone H3 were obtained from Santa Cruz Biotechnology (Santa Cruz, CA, USA). Oligonucleotide primers, SuperScript III First-Strand Synthesis System, and TRIzol Reagent were purchased from Invitrogen (Carlsbad, CA, USA). BCA protein assay kit, SYBR green PCR Master Mix, TurboFect Transfection Reagent, SuperSignal West Femto Maximum Sensitivity Substrate were obtained from Life Technologies/Thermo Fisher Scientific (Grand Island, NY, USA). Plasmids of pNF-κB-luc and pRL-TK were purchased from Addgene (Cambridge, MA, USA). Dual-luciferase assay kit was obtained from Promega (Madison, WI, USA). Triton X-100, polyvinylidene fluoride membranes were purchased from Bio-Rad (Hercules, CA, USA). mirVana PARIS Kit, TaqMan miRNA Reverse Transcription Kit, miRNA-specific stem-loop primers, TaqMan 2X Universal PCR Master Mix II, TaqMan miRNA-specific probe were obtained from Thermo Fisher Scientific. The synthetic spike-in control was purchased from QIAGEN (Hilden, Germany). Kits of AST, ALT, HDL-C, LDL-C, triglyceride TG, and TC were from Nanjing Jiancheng Bioengineering Institute (Nanjing, Jiangsu, China). α-Man was separated from the pericarps of *G. mangostana* [69], and its purity was determined to be ≥99.8% by high-performance liquid chromatography separation and ultraviolet detection (HPLC-UV).

## Supplementary Material

Supplementary Figures

Supplementary Tables
