# Supplementary material for: α-Mangostin remodels visceral adipose tissue inflammation to ameliorate age-related metabolic disorders in mice
Source: Aging (Albany NY). 2019 Dec 6;11(23):11084–110. doi: 10.18632/aging.102512 (PMC6932911; doi:10.18632/aging.102512)
Supplement: Supplementary Figures [file aging-11-102512-s002..pdf]

SUPPLEMENTARY FIGURES

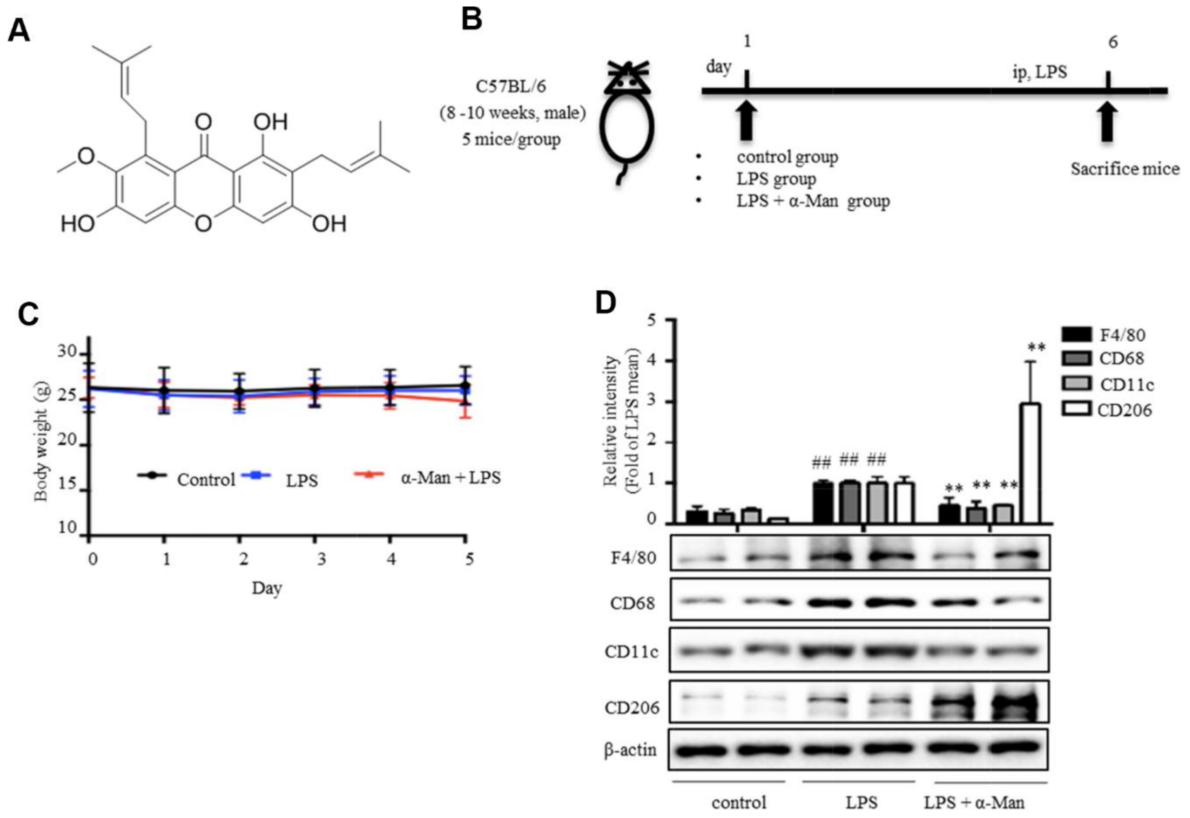

Supplementary Figure 1. Effects of α-mangostin in LPS-induced mice. (**A**) Chemical structure of α-Man. (**B**) The procedure of LPS-mediated acute inflammation mice model. (**C**) Effects of α-Man on body weight in LPS-treated mice. Data are expressed as means ± SD (*n* = 5).

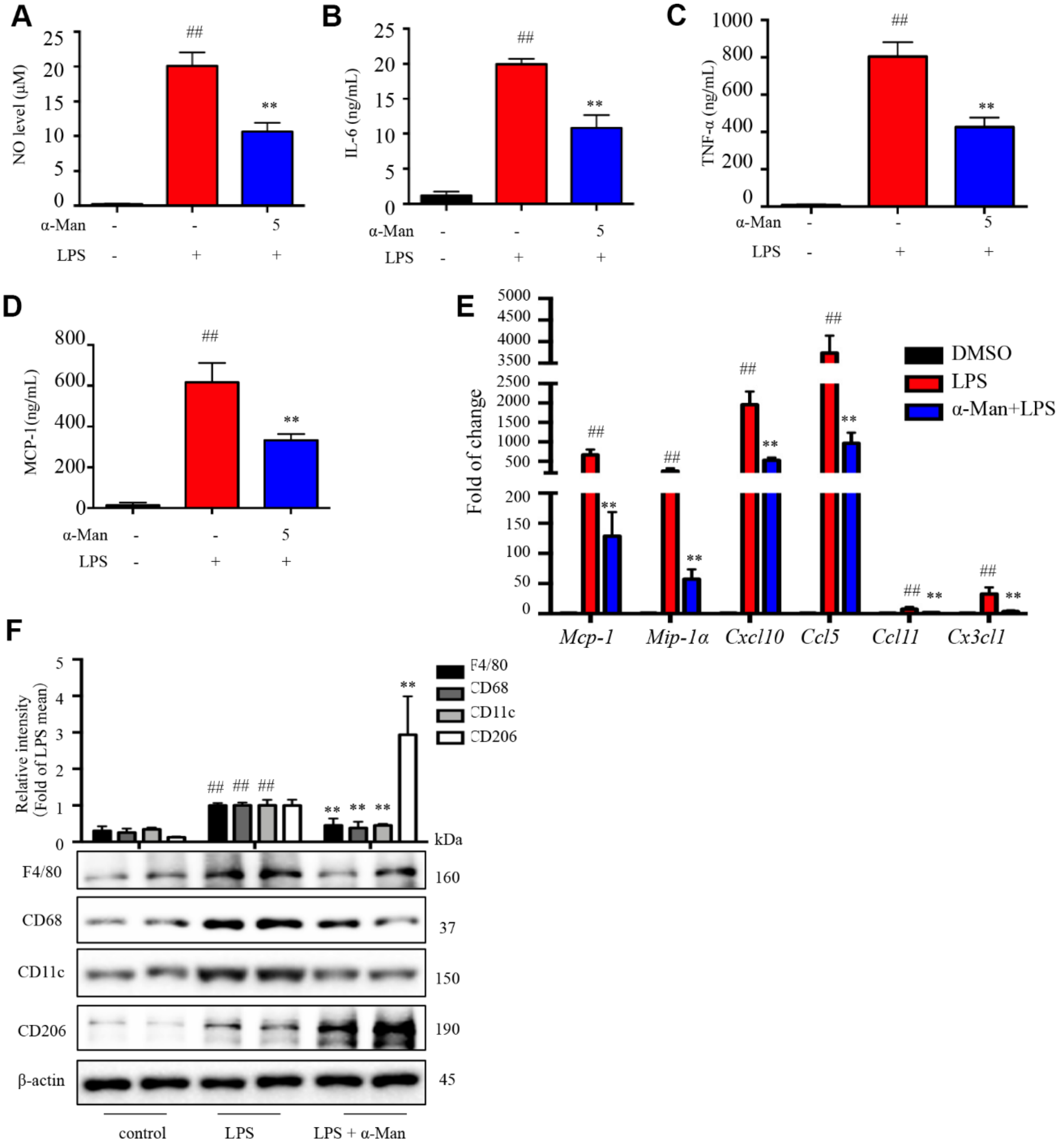

Supplementary Figure 2. α-Man suppresses cytokines and chemokines expression in LPS induced RAW264.7 macrophages. (**A**–**D**) RAW264.7 cells were seeded into 24-well plates (1 × 10^5 cells per well) and allowed to adhere for 24 hours. Then Cells were treated with α-Man (5 μM) in the presence of LPS (1 μg/mL) for 18 hours. NO production (**A**) was determined by Griess reagent. The levels of IL-6 (**B**), TNF-α (**C**) and MCP-1 (**D**) were determined by ELISA kit. (**E**) RAW264.7 macrophages were pre-treated with α-Man (5 μM) for 1 hour and then stimulated with LPS (1 μg/mL) for 6 hours. The mRNA levels of *MCP-1*, *MIP-1α*, *Cxcl10*, *Ccl5*, *Ccl11*, and *Cx3cl1* were analyzed by qRT-PCR and normalized with *18S*. (**F**) The expression of macrophage markers in adipose tissue from LPS-treated mice was determined by Western blots. β-Actin was used as internal loading control. Data are shown as means ± SD (n = 5). $^{\#\#}P < 0.01$, LPS vs. DMSO/control, $^{**}P < 0.01$, LPS + α-Man vs. LPS.

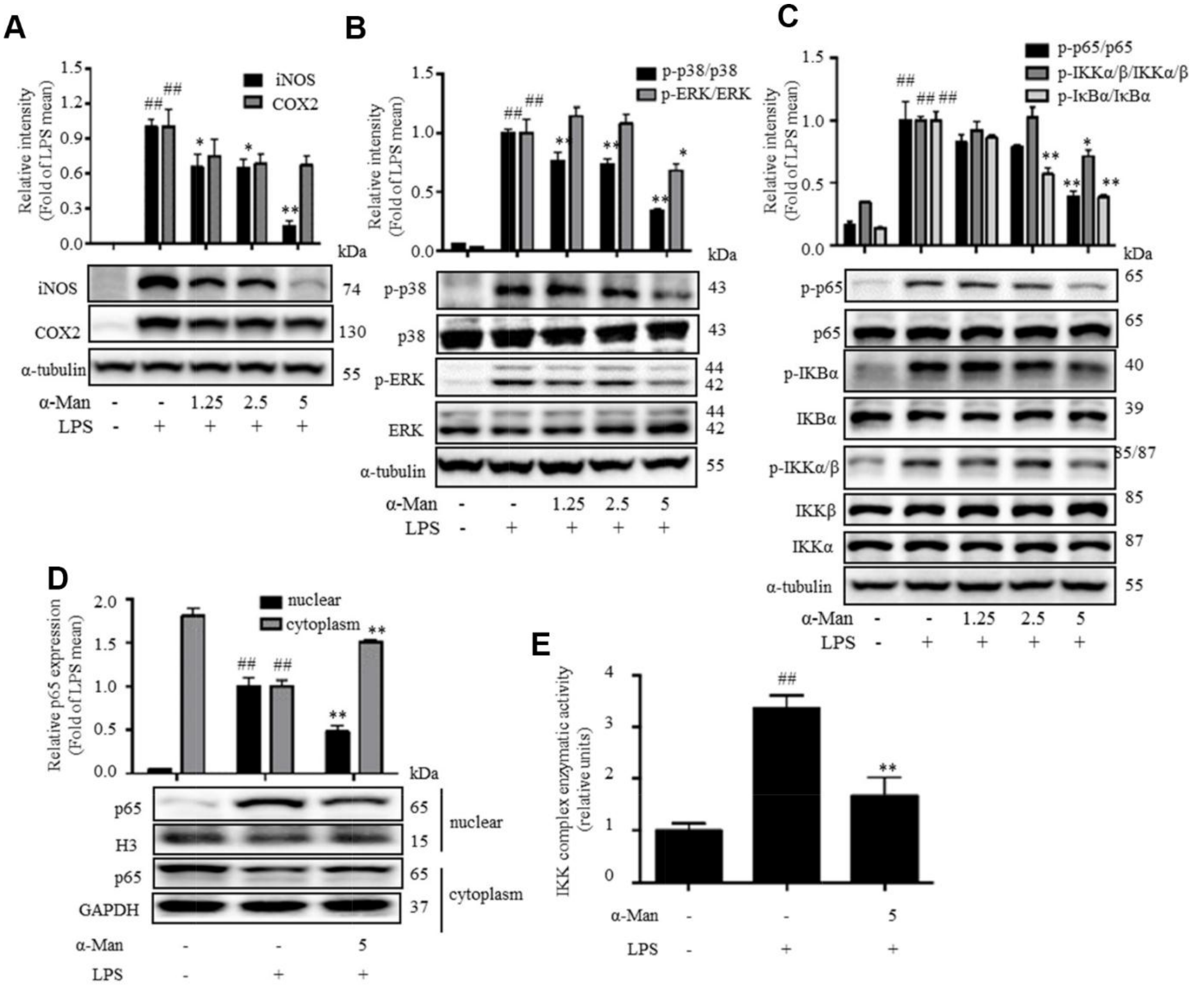

Supplementary Figure 3. α-Man inhibited inflammatory response in LPS stimulated RAW264.7 macrophages through MAPKs and NF-κB pathways. (**A**) The expression of iNOS and COX-2 protein was determined by Western blots. α-Tubulin was used as internal loading control. (**B**) The protein levels of p-ERK, ERK, p-p38 and p38 were determined by Western blots. α-Tubulin was used as internal loading control. (**C**) The protein levels of p-IKKα/β, IKKα, IKKβ, p-IκBα, IκBα, p-p65 and p65 were determined by Western blots. α-Tubulin was used as internal loading control. (**D**) The protein level of p65 in nuclear and cytoplasmic fractions was determined by Western blots. Histone 3 and GAPDH were used as internal loading controls. Data are normalized to the mean value of LPS group. (**E**) IKK kinase activity was measured and was expressed as % of the control without LPS and α-Man. Data are shown as means ± SD (*n* = 5). $^{##}P < 0.01$, LPS vs. DMSO, $^{*}P < 0.05$, $^{**}P < 0.01$, α-Man vs. LPS.

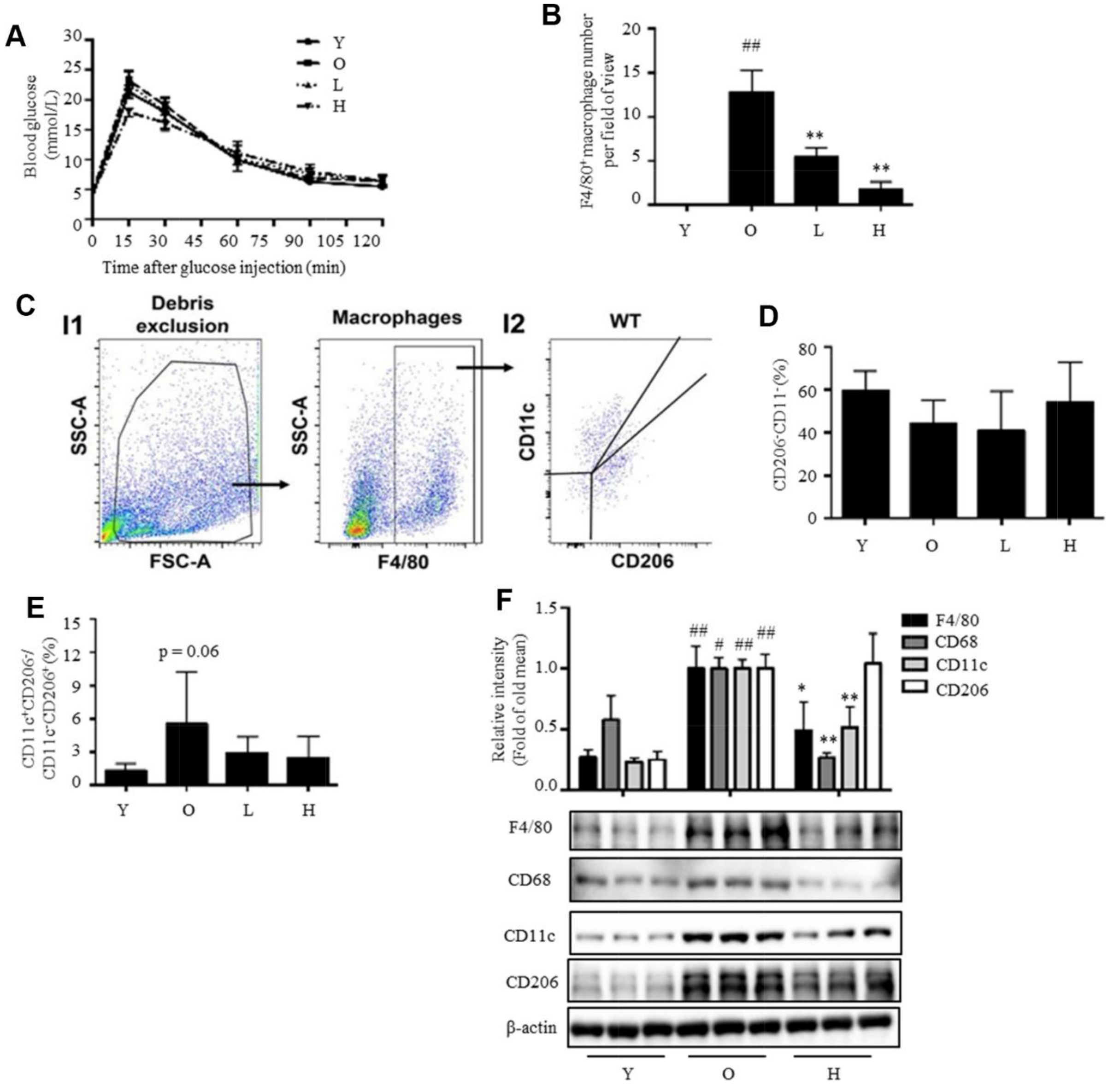

Supplementary Figure 4. Effects of α-Man in old mice. (**A**) GTT was performed in old mice after 6 weeks of intervention. (**B**) F4/80$^+$ macrophage number per field of view in Figure 5B. (**C**) Representative flow cytometry image analysis with gating and isotype controls. (I1) Debris exclusion and macrophage sorting. FSC-A, forward scatter area; SSC-A, side scatter area. (I2) Analyses of macrophages using CD11c and CD206. (**D**) ATM subtypes were quantified as a percentage of the total ATM population using flow cytometry. (**E**) The ratio of CD11c$^+$CD206$^-$ to CD11c$^-$CD206$^+$ ATMs. (**F**) The expression of macrophage markers in adipose tissue was determined by Western blots. β-Actin was used as internal loading control. Data are normalized to the mean value of old group. Data are expressed as means ± SD (*n* = 5). $^{\#}P < 0.05$, $^{\#\#}P < 0.01$, old mice vs. young mice, $^{*}P < 0.05$, $^{**}P < 0.01$, α-Man vs. old mice.

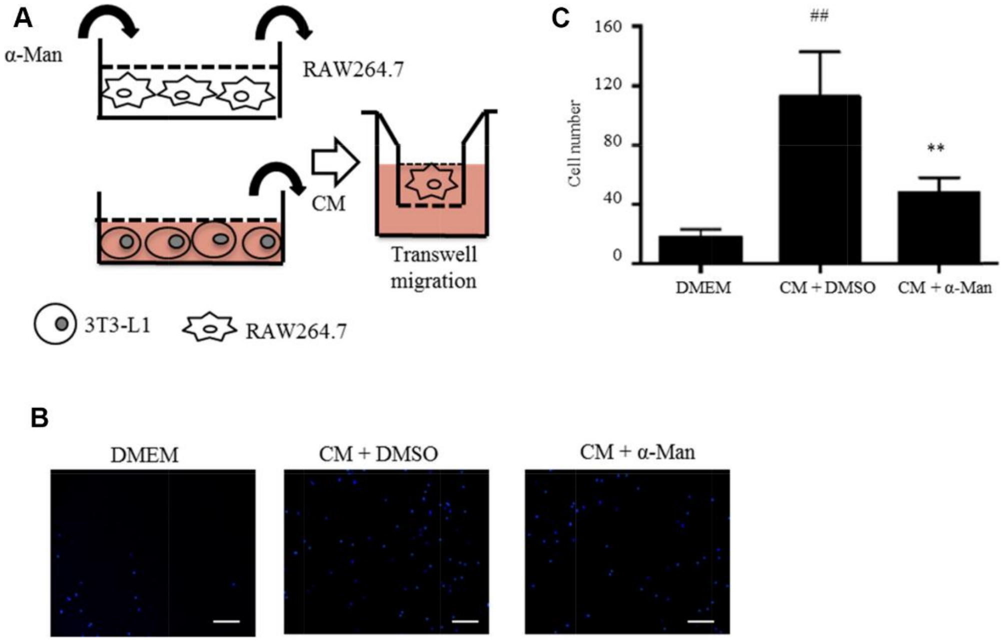

Supplementary Figure 5. Transwell assay of macrophage migration. (**A**, **B**) Schematic model and representative images of migrated macrophages. Scale bar = 200 µm. (**C**) RAW264.7 cells were treated with vehicle or α-Man (5 µM) for 4 hours and the detached cells were used for migration assay in the presence of DMEM or CM. Migrated RAW264.7 macrophages were visualized and quantitated by DAPI staining. Data are expressed as means ± SD (*n* = 5). $^{\#\#}P < 0.01$, CM + DMSO vs. DMEM, $^{**}P < 0.01$, CM + α-Man vs. CM + DMSO.

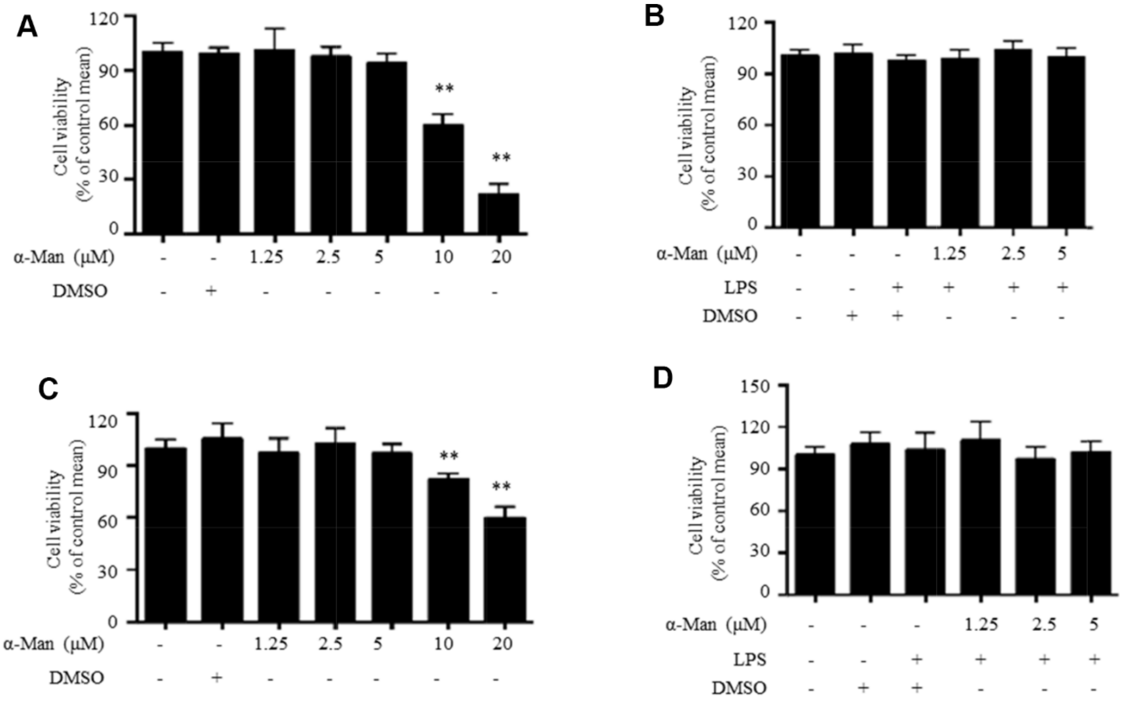

Supplementary Figure 6. Cytotoxicity of α-Man on cells. (**A**, **B**) The cytotoxicity of α-Man on RAW264.7 cells with or without LPS. RAW264.7 cells were treated with α-Man ranging from 1.25 to 5 µM in the presence or absence of LPS (1 µg/mL) for 24 hours. (**C**, **D**) The cytotoxicity of α-Man on BMDMs with or without LPS. BMDMs were treated with α-Man ranging from 1.25 to 5 µM in the presence or absence of LPS (1 µg/mL) for 24 hours. Cell viability was determined by MTT assay. Data are normalized to the mean value of control group. Data are expressed as means ± SD (*n* = 6). $^{**}P < 0.01$, α-Man vs. control.
