# Supplementary material for: α-Mangostin remodels visceral adipose tissue inflammation to ameliorate age-related metabolic disorders in mice
Source: Aging (Albany NY). 2019 Dec 6;11(23):11084–110. doi: 10.18632/aging.102512 (PMC6932911; doi:10.18632/aging.102512)
Supplement: Supplementary Tables [file aging-11-102512-s001..pdf]

SUPPLEMENTARY TABLES

Supplementary Table 1. Effect of α-Man on body weight of old mice.

Week	Body weight (g)			
	Y	O	L	H
0	24.32±2.31	40.34±5.37$^{\#\#}$	37.92±5.32	38.94±4.79
1	24.66±2.41	39.26±5.42$^{\#\#}$	34.52±4.58	34.2±4.71
2	24.66±2.48	39.22±5.37$^{\#\#}$	34.18±4.27	33.68±4.29
3	25.06±2.10	39.04±5.40$^{\#\#}$	34.34±3.87	32.66±6.39
4	26.46±1.63	39.84±4.22$^{\#\#}$	35.08±3.84	31.50±4.88**
5	26.54±1.53	38.30±3.63$^{\#\#}$	34.72±3.68	31.14±4.40*
6	25.76±1.80	37.22±4.03$^{\#\#}$	34.56±3.71	30.28±4.19*
7	26.44±2.14	37.02±4.01$^{\#\#}$	33.58±3.66	30.58±3.16*
8	26.00±2.12	36.50±3.54$^{\#\#}$	32.42±3.15	30.48±3.06*

$^{\#\#}P < 0.01$ old mice vs. young mice. $^{*}P < 0.05$, $^{**}P < 0.01$ α-Man vs. old mice.

Supplementary Table 2. List of oligonucleotide primer pairs used in qRT-PCR.

Gene	Forward	Reverse
18S	AGCCTGCGGCTTAATTTGAC	CAACTAAGAACGGCCATGCA
Arg-1	CTCCAAGCCAAAGTCCTTAGAG	AGGAGCTGTCATTAGGGACATC
Ccl5	TGCAGAGGACTCTGAGACAGC	GAGTGGTGTCCGAGCCATA
Ccl11	AGAGCTCCACAGCGCTTCT	GCAGGAAGTTGGGATGGA
Cd11c	CTGGATAGCCTTTCTTCTGCTG	GCACACTGTGTCCGAACTC
Cd206	CAGGTGTGGGCTCAGGTAGT	TGTGGTGAGCTGAAAGGTGA
Cd68	CCTTATGGACAGCTTACCTTTGG	CTGAGCAGCCTGTAGCCTTAGAG
Cox-2	CCACCTCTGCGATGCTCTTC	CATTCCCCACGGTTTTGACATG
Cx3cl1	CATCCGCTATCAGCTAAACCA	CAGAAGCGTCTGTGCTGTGT
Cxcl10	GCTGCCGTCATTTTCTGC	TCTCACTGGCCCGTCATC
F4/80	CTTTGGCTATGGGCTTCCAGTC	GCAAGGAGGACAGAGTTTATCGTG
Ifn-γ	GTCATTGAAAGCCTAGAAAGTCTGA	CTGTGGGTTGTTGACCTCAAACT
Il-1β	TGTTCTTTGAAGTTGACGGACCC	TCATCTCGGAGCCTGTAGTGC
Il- 6	CCAGAGATACAAAGAAATGATGG	ACTCCAGAAGACCAGAGGAAAT
iNos	CCAAGCCCTCACCTACTTCC	CTCTGAGGGCTGACACAAGG
Mcp-1	CAACTCTCACTGAAGCCAGCTC	TAGCTCTCCAGCCTACTCATTGG
Mip-1α	CTTCTCTGTACCATGACACTCTGC	ATTCAGTTCCAGGTCAGTGATGTAT
Tnf-α	GAGAAAGTCAACCTCCTCTCTG	GAAGACTCCTCCCAGGTATATG

Supplementary Table 3. List of miRNA primers used in qRT-PCR.

miRNA	Company	Catalog number
U6 snRNA RT-PCR primer	Thermo Fisher Scientific	4427975(ID#001973)
miR-155 RT-PCR primer	Thermo Fisher Scientific	4427975(ID#002571)
Cel-miR-39	Thermo Fisher Scientific	4427975(ID#000200)

Supplementary Table 4. Antibodies used for Western blot and flow cytometry.

Antibody	Source	Vendor	Catalog No.
anti-phospho-JNK (Thr183/Tyr185)	Mouse	Santa Cruz Biotechnology	sc-6254
anti-JNK	Rabbit	Santa Cruz Biotechnology	sc-571
anti-phospho-p38 (Thr180/Tyr182)	Rabbit	Cell Signaling Technology	#4511
anti-p38	Rabbit	Cell Signaling Technology	#8690
anti-phospho-ERK (Thr202/Tyr204)	Rabbit	Cell Signaling Technology	#4370
anti-ERK	Rabbit	Cell Signaling Technology	#4695
anti-phospho-p65 (Ser536)	Rabbit	Cell Signaling Technology	#3033
anti- p65	Rabbit	Cell Signaling Technology	#8242
anti-phospho-IKKα/β (Ser176/180)	Rabbit	Cell Signaling Technology	#2697
anti-IKKα	Mouse	Cell Signaling Technology	#11930
anti-IKKβ	Rabbit	Cell Signaling Technology	#8943
anti-phospho-IκBα (Ser32)	Rabbit	Cell Signaling Technology	#2859
anti-IκBα	Mouse	Cell Signaling Technology	#4814
anti-iNOS	Rabbit	Cell Signaling Technology	#13120
anti-COX2	Rabbit	Cell Signaling Technology	#12282
anti-SIRT3	Rabbit	Cell Signaling Technology	#5490
anti-AKT	Rabbit	Santa Cruz Biotechnology	sc-8312
anti- phospho-AKT (Ser473)	Rabbit	Santa Cruz Biotechnology	sc-7985-R
anti-perilipin	Rabbit	Cell Signaling Technology	#9349
anti-CD68	Rabbit	ABclonal	A6554
anti-F4/80	Mouse	eBioscience	12-4801-82
anti-F4/80	Mouse	Santa Cruz Biotechnology	sc-377009
anti-CD11c	Mouse	BD Bioscience	557400
anti-CD11c	Rabbit	ABclonal	A1508
anti-CD206	Mouse	BioLegend	141708
anti-CD206	Rabbit	Abcam	ab64693
α-tubulin	Mouse	Santa Cruz Biotechnology	sc-8035
β-actin	Rabbit	Cell Signaling Technology	#8457
GAPDH	Rabbit	Santa Cruz Biotechnology	sc-25778
Histone H3	Rabbit	Santa Cruz Biotechnology	sc-10809
